# CK20 versus AMACR and p53 immunostains in evaluation of Urothelial Carcinoma in Situ and Reactive Atypia

**DOI:** 10.1186/s13000-020-00984-2

**Published:** 2020-05-26

**Authors:** Daniel J. Neal, Mahul B. Amin, Steven C. Smith

**Affiliations:** 1 Department of Pathology, Virginia Commonwealth University School of Medicine, PO Box 980662, Richmond, VA 23298 USA; 2 Department of Pathology and Laboratory Medicine, University of Tennessee Health Sciences Center, Memphis, TN 38163 USA; 3 Division of Urology, Department of Surgery, Virginia Commonwealth University School of Medicine, PO Box 980662, Richmond, VA 23298 USA

**Keywords:** Urothelial carcinoma in situ, Cytokeratin 20, Alpha-methylacyl-CoA racemase

## Abstract

Ancillary testing with immunohistochemistry has shown recent promise in the workup of equivocal bladder lesions. We read with interest the recent findings of Alston et al., who assessed the diagnostic utility of alpha-methylacyl-CoA racemase (AMACR) in comparison to cytokeratin 20 (CK20) in evaluation of atypia in challenging flat urothelial lesions in the differential between carcinoma in situ (CIS) and reactive atypia. AMACR was reported to be a somewhat more specific but less sensitive marker for CIS than CK20, though showing weaker intensity. Spurred by their report, with the knowledge that we had consistently and consecutively performed AMACR, CK20, and p53 on flat urothelial lesions challenging enough to reach intradepartmental consensus, we performed a retrospective review. Similarly, we found that AMACR was less sensitive (80%) and more specific (100%) than CK20, with the same caveat of less staining intensity. Additionally, our p53 review identified a significant rate (~ 27%) of equivocal/non-informative findings. Taken together, our experience in this consecutive cohort confirms the impression of Alston et al. regarding the utility and challenges of AMACR use, while highlighting challenges with p53, which we plan to use more sparingly prospectively.

## Introduction

Distinguishing between urothelial carcinoma in situ (CIS) and reactive cellular atypia in bladder specimens can prove challenging [1, 2], requiring careful correlation with clinical and cytologic features [3, 4]. Though recent studies have shown the overexpression of cytokeratin 20 (CK20), p53 and even alpha-methylacyl-CoA racemase (AMACR) in CIS [5–7], few studies have evaluated each marker’s performance in consecutive clinical cases. Recently Alston et al. reviewed 52 cases in which immunohistochemistry (IHC) for CK20 and AMACR was performed on cases with final diagnoses consisting of CIS or non-neoplastic/reactive [8]. They additionally identified and stained 20 unequivocal cases of CIS in which prior IHC was not performed. In their experience, AMACR had a sensitivity of 73% and specificity of 97%, while CK20 had a sensitivity of 95% and specificity of 80%. CK20 showed stronger, more consistent staining, while weak, surface positivity was seen in both among non-neoplastic cases. Based on its higher sensitivity and consistent, strong staining pattern, CK20 was suggested as perhaps a better suited ancillary test for distinguishing CIS versus reactive atypia. Given that in our department we had as a standard procedure ordered AMACR and CK20, as well as p53, on cases thought to require consensus opinion in recent years, we performed herein a retrospective review for comparison.

## Methods

We retrospectively reviewed 31 cases from 29 different patients from 2014 to 2019 (CK20 clone SP33; AMACR clone SP116; p53 clone DO-7; all predilute and performed on a Ventana Benchmark XT). All slides were re-reviewed, including original H&E and IHC stains, for confirmation of diagnoses and exclusion of processes such as atypia related to BK virus [9]. Clinicopathologic features were tabulated. Staining for CK20 and AMACR was evaluated similarly to Alston et al. [8] as negative (focal/luminal expression), patchy (partial positive > 1/3 but ≤2/3 of the urothelium), or diffuse (> 2/3 thickness), with intensity scored as negative (0), weak (1+), or strong (2+). Evaluation of nuclear p53 expression was scored as wild-type (scattered, usually weak-moderate) mutant-type (strong consistent staining or complete absence of staining in atypical cells) or equivocal (any other pattern observed).

## Results

A total of 31 samples from 29 patients (17 M; 12 F) were reviewed, with median age of 65 years. Specimens including 16 bladder biopsies, 11 transurethral resections (TURBTs), 2 resections including one partial cystectomy and one pelvic exenteration, 1 ureter biopsy, and one transurethral resection of the prostate (TURP). Final diagnoses included 15 CIS and 15 reactive urothelium (Table 1), with an additional case finally diagnosed as high-grade squamous intraepithelial lesion (HSIL) of gynecologic origin. Of cases with a final diagnosis of CIS, 14 of 15 (93%) showed positive staining for CK20, compared to 12 of 15 (80%) positive with AMACR. However, of cases diagnosed as reactive atypia, 2 of 15 (13%) showed positive staining for CK20, compared to 0 of 15 (0%) for AMACR. As expected, CK20 showed greater staining in non-neoplastic umbrella cells. AMACR staining was statistically significantly less intense but not less diffuse than CK20 (*p* = 0.02 and *p* = 0.07, respectively, paired Wilcoxon). Across all specimens, p53 showed a significant rate of equivocal/non-informative staining (27%), disproportionately (63%) among cases diagnosed as CIS.

**Table 1:** CK20, AMACR, and p53 staining in Intradepartmental Consensus Urothelial Lesions

		CK20 Proportion			CK20 Intensity			AMACR Proportion			AMACR Intensity			p53 Pattern^**a**^		
	N (%)	–	±	+	0+	1+	2+	–	±	+	0+	1+	2+	Wild type	Mutant	Equivocal
**Reactive**	15	13 (87)	1 (7)	1 (7)	13 (87)	1 (7)	1 (7)	15 (100)	0	0	15 (100)	0	0	12 (80)	0	3 (20)
**CIS**	15	1 (7)	4 (27)	10 (67)	1 (7)	4 (27)	10 (67)	3 (20)	5 (33)	7 (47)	3 (20)	8 (53%)	4 (27)	1 (7)	9 (60)	5 (33)

*Abbreviations: CK20* cytokeratin 20, *AMACR* alpha-methylacyl-CoA racemase;

Staining Proportion: -, negative; ±, patchy; +, diffuse

Staining Intensity: 0+ negative; 1+ weak; 2+ strong

^a^ Please see Methods for description of scoring pattern

## Discussion

**Fig. 1:**
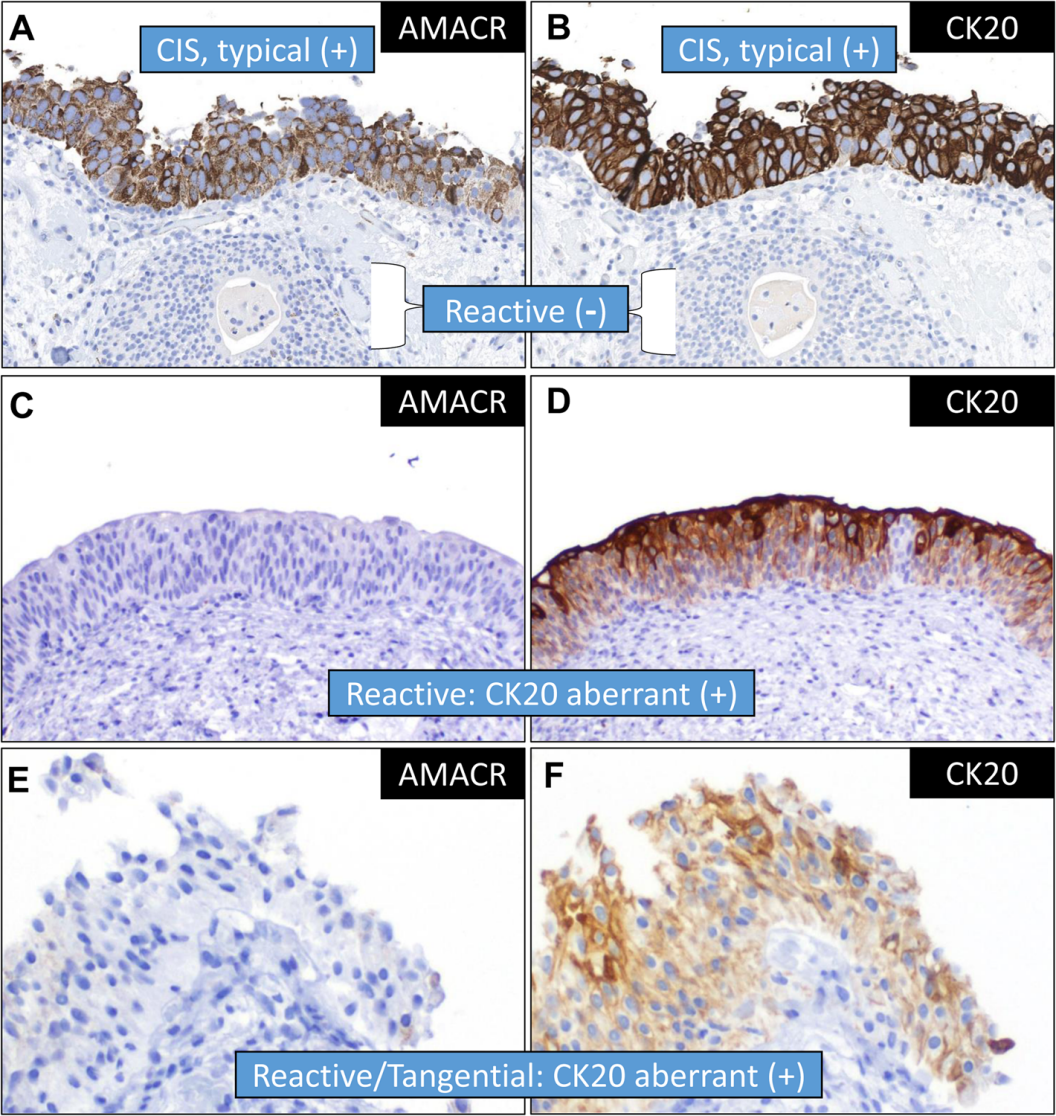
The most characteristic pattern of staining in urothelial carcinoma in situ (CIS) was of diffuse, full thickness (+) staining for AMACR (**a**) and CK20 (**b**). AMACR typically demonstrated reduced intensity and a granular cytoplasmic pattern, as compared to CK20; additionally, reactive urothelium in the nest of cystitis cystica underlying the surface CIS demonstrates expected negativity for both markers. Two examples of the challenges in the use of these markers where AMACR added utility included one case of reactive urothelium showing negative AMACR (**c**) but aberrantly positive CK20, relatively strong in most of the thickness of the urothelium (**d**). Additionally, we noted examples of small biopsies where AMACR was negative (**e**) but CK20 appeared positive (**f**), even if relatively weakly so, due in part to staining of normal umbrella cells sectioned tangentially en face

In conjunction with the findings from Alston et al., our results support the use of CK20 and AMACR IHC in equivocal urothelial lesions in the differential between CIS and reactive atypia (Fig. 1a-b). We confirm the finding of AMACR showing marginally less sensitivity, more specificity (one example in Fig. 1c-d), but less intensity overall. Anecdotally, we also saw cases with areas of tangential sectioning of umbrella cells simulating CK20 positivity but where AMACR was negative (Fig. 1e-f) due to its essential lack of expression in non-neoplastic urothelium. This may represent one relative advantage of AMACR; in any case, given our significant rate of equivocal findings we plan to substitute AMACR for p53 going forward. While the reasons for CK20 and AMACR expression is CIS remain unknown [10], the addition of AMACR to the diagnostic armamentarium in this setting is welcome.

## Data Availability

The datasets used and analysed during the current study are available from the corresponding author on reasonable request.

## Abbreviations

AMACR: alpha-methylacyl-CoA racemase
CK20: cytokeratin 20
IHC: immunohistochemistry
